# Distinct CSF biomarker-associated DNA methylation in Alzheimer’s disease and cognitively normal subjects

**DOI:** 10.21203/rs.3.rs-2391364/v1

**Published:** 2023-02-21

**Authors:** Wei Zhang, Juan I. Young, Lissette Gomez, Michael A. Schmidt, David Lukacsovich, Achintya Varma, X. Steven Chen, Eden R. Martin, Lily Wang

**Affiliations:** University of Miami, Miller School of Medicine; Dr. John T Macdonald Foundation, University of Miami, Miller School of Medicine; University of Miami Miller School of Medicine; Dr. John T Macdonald Foundation, University of Miami, Miller School of Medicine; University of Miami, Miller School of Medicine; University of Miami Miller School of Medicine; University of Miami, Miller School of Medicine; Dr. John T Macdonald Foundation, University of Miami, Miller School of Medicine; University of Miami, Miller School of Medicine

**Keywords:** DNA methylation, CSF biomarkers, Alzheimer’s disease

## Abstract

**Background:**

Growing evidence has demonstrated that DNA methylation (DNAm) plays an important role in Alzheimer’s disease (AD) and that DNAm differences can be detected in the blood of AD subjects. Most studies have correlated blood DNAm with the clinical diagnosis of AD in living individuals. However, as the pathophysiological process of AD can begin many years before the onset of clinical symptoms, there is often disagreement between neuropathology in the brain and clinical phenotypes. Therefore, blood DNAm associated with AD neuropathology, rather than with clinical data, would provide more relevant information on AD pathogenesis.

**Methods:**

We performed a comprehensive analysis to identify blood DNAm associated with cerebrospinal fluid (CSF) pathological biomarkers for AD. Our study included matched samples of whole blood DNA methylation, CSF Aβ_42_, phosphorylated tau_181_ (pTau_181_), and total tau (tTau) biomarkers data, measured on the same subjects and at the same clinical visits from a total of 202 subjects (123 CN or cognitively normal, 79 AD) in the Alzheimer’s Disease Neuroimaging Initiative (ADNI) cohort. To validate our findings, we also examined the association between premortem blood DNAm and postmortem brain neuropathology measured on a group of 69 subjects in the London dataset.

**Results:**

We identified a number of novel associations between blood DNAm and CSF biomarkers, demonstrating that changes in pathological processes in the CSF are reflected in the blood epigenome. Overall, the CSF biomarker-associated DNAm is relatively distinct in CN and AD subjects, highlighting the importance of analyzing omics data measured on cognitively normal subjects (which includes preclinical AD subjects) to identify diagnostic biomarkers, and considering disease stages in the development and testing of AD treatment strategies. Moreover, our analysis revealed biological processes associated with early brain impairment relevant to AD are marked by DNAm in the blood, and blood DNAm at several CpGs in the DMR on *HOXA5* gene are associated with pTau_181_ in the CSF, as well as tau-pathology and DNAm in the brain, nominating DNAm at this locus as a promising candidate AD biomarker.

**Conclusions:**

Our study provides a valuable resource for future mechanistic and biomarker studies of DNAm in AD.

## Introduction

Late-onset Alzheimer’s disease (LOAD), affecting about 1 in 9 people 65 years and older in the US^1^, has become a major public health problem and one of the most financially costly diseases^2^. Clinically, Alzheimer’s disease (AD) is characterized by progressive deterioration of cognitive functions, eventually leading to a lack of ability to carry out even the simplest tasks, which places significant emotional, financial, and physical burdens on caregivers. Growing evidence has demonstrated that DNA methylation (DNAm), a widely studied epigenetic mechanism that modifies gene expression without changing the underlying DNA sequences, plays an important role in AD^3–5^. In particular, recent studies have identified and replicated a number of DNAm loci in the brain (e.g., *ANK1, RHBDF2,* and *HOXA*) that are robustly associated with AD neuropathology^6–10^. Encouragingly, it has become increasingly evident that DNAm differences can also be detected in the blood of AD subjects^11–16^. Most recently, our meta-analysis of two large clinical AD datasets revealed a number of DNAm loci in the blood significantly associated with AD diagnosis^17^.

Given that it still is not practical to obtain methylation levels in brain tissues from living human subjects, most studies have correlated blood DNAm with AD diagnosis. However, as the pathophysiological process of AD can begin many years before the onset of clinical symptoms^18,19^, there is often disagreement between neuropathology and clinical phenotypes^20,21^. Currently, there is still limited knowledge on the association of blood DNAm and changes in AD neuropathology.

CSF biomarkers are well-established AD endophenotypes, and their abnormality is predictive of the onset and progression of AD^22–27^. Encouragingly, premortem CSF biomarker values also correlate significantly with neuropathology scores measured on postmortem brain samples^28^. The hallmark of AD is the accumulation of aggregated amyloid and tau proteins in the brain. Under the AT(N) framework^29,30^, cerebrospinal fluid (CSF) levels of Aβ_42_, phosphorylated tau at threonine 181 (pTau_181_), and total tau corresponds to the accumulation of Aβ plaque (A), fibrillary tau (T), and non-disease-specific neurodegeneration (N), respectively.

In this study, we performed a comprehensive analysis to identify blood DNA methylation associated with CSF biomarkers in the Alzheimer’s Disease Neuroimaging Initiative (ADNI) cohort. In addition to a greater understanding of the regulatory changes associated with different pathological disease-associated processes in living individuals, compared to previous analyses that used clinical AD diagnosis as the endpoint, we also expected this analysis of CSF biomarkers, which are quantitative measurements, would help with improving statistical power. To prioritize the significant CSF biomarker-associated DNAm, we performed several integrative analyses that additionally included gene expression and genetics data, as well as a validation analysis which analyzed the London dataset with both premortem blood DNAm and postmortem brain neuropathology measured on a group of 69 subjects. Results from this study provide an improved understanding of the epigenetics underlying inter-individual variations in various pathological pathways involved in AD.

## Methods

### Study dataset

The ADNI is a longitudinal study that aims to define the progression of AD^31^. To create a dataset with independent samples, we only analyzed the last visit data of each subject from the longitudinal ADNI study. Our blood sample dataset included 202 DNA methylation samples (123 cognitively normal (CN) samples and 79 AD samples) with available CSF biomarkers information (Aβ_42_, phosphorylated tau_181_, and total tau) measured on the same subject at the same clinical visit in the ADNI study. To avoid the inclusion of early-onset AD subjects, only subjects older than 65 years of age were included. The study datasets can be accessed from the ADNI study website (adni.loni.usc.edu). In Table 1, sample characteristics for the CN and AD groups were compared using Fisher’s exact test for categorical variables and the Wilcoxon Rank Sum test for continuous variables.

### Pre-processing of DNA methylation data

The DNA methylation samples were measured with the Illumina HumanMethylation EPIC beadchip, which includes more than 850,000 CpGs. Supplementary Table 1 shows the number of probes and samples removed at each step of quality control (QC). For the QC of probes, we first selected probes with a detection *P*-value < 0.01 in every sample. A small detection *P*-value (i.e., *P*-value < 0.01) indicates a significant difference between the signals in the probe and the background noise. Next, using the rmSNPandCH function from the DMRcate R package, we removed probes that are cross-reactive^32^, located close to single nucleotide polymorphism (SNPs) (i.e., an SNP with minor allele frequency (MAF) ≥ 0.01 was present in the last five base pairs of the probe), or located on X or Y chromosomes. QC for samples included restricting our analysis to samples with good bisulfite conversion efficiency (i.e., ≥ 85%). In addition, principal component analysis (PCA) was used to remove the outlier samples. Specifically, PCA was performed using the 50,000 most variable CpGs, and we selected samples within ± 3 standard deviations from the mean of the first PC and second PC. Finally, we excluded samples without matching clinical or CSF biomarkers information.

The quality-controlled methylation samples were then subjected to the QN.BMIQ normalization procedure^33^, which included between-array quantile normalization (QN) followed by within-array β-mixture quantile normalization (BMIQ)^34^. For the QN step, we used the betaqn function in the wateRmelon R package (version 1.99.1) to remove systematic effects between samples. For the BMIQ procedure, which is also implemented in the wateRmelon R package, the distributions of beta values measured by type 1 and type 2 design probes were normalized within each Illumina array.

Immune cell type proportions, including B lymphocytes, natural killer cells, CD4 + T lymphocytes, monocytes, and granulocyte, were estimated using the EpiDISH R package (version 2.12.0) ^35^. Here, the granulocyte proportions were computed as the sum of neutrophils and eosinophils proportions since neutrophils and eosinophils are classified as granular leukocytes, as previously described ^36,37^.

### CSF Biomarkers

We obtained information for CSF biomarkers (A*β*_42_, pTau_181_, and tTau), which were measured by Roche Elecsys immunoassay, from the “UPENNBIOMK9.CSV” file at the ADNI website (adni.loni.usc.edu). Standardized CSF biomarkers values were computed by log (base 2)-transformation followed by centering using the study means, as in previous analyses of CSF biomarkers^38,39^.

### Identification of CSF biomarker-associated CpGs

To assess the associations between CSF biomarkers (A*β*_42_, pTau_181_, and tTau) and DNA methylation, we fitted the following linear regression model (Model 1) to CN and AD samples separately: standardized CSF biomarker ~ methylation.beta + age + methylation plate + sex + APOE4 + years of education + smoking history + immune cell-type proportions (B, NK, CD4T, Mono, Gran).

We also compared the effects of methylation-to-CSF biomarker associations in CN samples and AD samples, by fitting the following model (Model 2) to combined CN and AD samples: standardized CSF biomarker ~ methylation.beta + diagnosis + methylation.beta × diagnosis + age + methylation plate + sex + APOE4 +years of education + smoking history + immune cell-type proportions (B, NK, CD4T, Mono, Gran). Significant methylation.beta × diagnosis interaction effect corresponds to a significant difference in methylation-to-CSF biomarker associations in the CN samples and AD samples.

### Inflation Assessment and correction

We estimated genomic inflation factors (lambda values) using both the conventional approach^40^ and the bacon method^41^, which is specifically proposed for a more accurate assessment of inflation in EWAS. Supplementary Table 2 shows the estimated inflation and bias of the test statistics from Model 1 described above. Specifically, lambda values (λ) by the conventional approach ranged from 0.719 to 1.096, and lambdas based on the bacon approach (λ.bacon) ranged from 0.863 to 1.019. The estimated bias ranged from − 0.097 to 0.117. Genomic correction using the bacon method^41^, as implemented in the *bacon* R package, was then applied to obtain bacon-corrected effect sizes, standard errors, and *P*-values for each dataset to obtain a more accurate estimate of statistical significance. After bacon correction, the estimated bias ranged from − 0.002 to 0.002, the estimated inflation factors ranged from λ = 0.967 to 1.042, and λ.bacon ranged from 0.974 to 1.000.

For each CSF biomarker, we considered CpGs with a false discovery rate (FDR) ≤ 0.05 as statistically significant. Given the modest number of samples with both DNA methylation and CSF biomarker measurements, we expected our analysis to be underpowered. Therefore, based on our experiences and previous studies in the analysis of EWAS measured in blood^37,42,43^, we also prioritized CpGs with suggestive significance at the pre-specified significance threshold *P*-value < 1 × 10^−5^.

### Differentially methylated regions (DMR) analysis

To identify the differentially methylated regions associated with CSF biomarkers, we used the comb-p software^44^. Briefly, comb-p takes single CpG *P*-values and locations of the CpG sites to scan the genome for regions enriched with a series of adjacent low *P*-values. In our analysis, we used the bacon-corrected *P*-values from Model 1 above as the input, and the parameter setting --seed 0.05 and --dist 750 (a *P*-value of 0.05 is required to start a region and extend the region if another *P*-value was within 750 base pairs), which was shown to have optimal statistical properties in our previous comprehensive assessment of the comb-p software^45^. As comb-p uses the Sidak method to correct *P*-values for multiple comparisons, we considered DMRs with Sidak-adjusted *P*-value < 0.05 as significant. To further reduce false positives, we imposed two additional criteria in our final selection of DMRs: (1) DMRs with nominal *P*-value < 1 × 10^− 5^; (2) All CpGs within the DMR have a consistent direction of change in estimated effect sizes from Model 1 described above.

### Functional annotation of significant methylation associations

The significant methylation at individual CpGs and DMRs was annotated using both the Illumina (UCSC) gene annotation, and Genomic Regions Enrichment of Annotations Tool (GREAT) software which associates genomic regions to target genes^46^. To assess the overlap between our significant CpGs and DMRs (CpG or DMR location +/−250bp) with enhancers, we used enhancer–gene maps generated from 131 human cell types and tissues described in Nasser et al. (2021)^47^ (https://www.engreitzlab.org/resources/). Specifically, we selected enhancer-gene pairs with “positive” predictions from the ABC model, which included only expressed target genes, did not include promoter elements, and had an ABC score higher than 0.015. In addition, we also required that the enhancer-gene pairs be identified in cell lines relevant to this study (https://github.com/TransBioInfoLab/AD-meta-analysis-blood/blob/main/code/annotations/).

### Pathway analysis

To identify biological pathways enriched with CSF biomarker-associated DNA methylation, we used the methylRRA function in the methylGSA R package^48^ (version 1.14.0). The pathway analyses were performed separately for each of the three CSF biomarkers, and the most significant *P*-value among the 3 *P*-values (one for each CSF biomarker) was then selected as the final *P*-value for each pathway. In each analysis, we used the bacon-corrected *P*-values from Model 1 described above as the input for methylGSA. Briefly, methylGSA first computes a gene-wise *ρ* value by aggregating *P*-values from multiple CpGs mapped to each gene. Next, the different number of CpGs on each gene is adjusted by Bonferroni correction. Finally, a Gene Set Enrichment Analysis^49^ (in pre-rank analysis mode) is performed to identify pathways enriched with significant CSF-associated DNAm. We analyzed pathways in the KEGG^50^ and REACTOME^51^ databases. Because of the relatively smaller number of gene sets being tested, a 25% FDR significance threshold, instead of the conventional 5% FDR, was suggested to be the default significance threshold for GSEA (https://software.broadinstitute.org/cancer/software/gsea/wiki/index.php/FAQ). Therefore, we considered pathways with FDR < 0.25 as statistically significant.

### Integrative methylation-to-gene expression analysis

To evaluate the DNA methylation effect on the gene expression of nearby genes, we analyzed matched gene expression (Affymetrix Human Genome U 219 array) and DNA methylation (EPIC array) data from 263 independent subjects in the ADNI study (adni.loni.usc.edu). To reduce the effect of potential confounding, when testing methylation-to-gene expression associations, we first adjusted age at visit, sex, immune cell-type proportions (for B lymphocytes, natural killer cells, CD4 + T lymphocytes, monocytes, granulocytes), batch effects, number of APOE4 alleles, smoking history, and years of education in both DNA methylation and gene expression levels separately and extracted residuals from the linear models. Immune cell-type proportions were estimated using the R packages EpiDISH ^35^ and Xcell^52^ (https://github.com/dviraran/xCell) for DNA methylation and gene expression data, respectively. A separate linear model was then used to test for the association between methylation residuals and gene expression residuals, separately for CN and AD samples. For the analysis of DMRs, we summarized each DMR by the median methylation value of all CpGs mapped within the DMR, extracted residuals, and then fitted the linear model described above, by replacing the methylation value for the CpG with the median methylation value for the DMR.

### Correlation and overlap with genetic susceptibility loci

We searched mQTLs using the GoDMC database^53^, which was downloaded from http://mqtldb.godmc.org.uk/downloads. To select significant blood mQTLs in GoDMC, we used the same criteria as the original study^53^, that is, considering a cis *P*-value smaller than 10^− 8^ and a trans-*P*-value smaller than 10^− 14^ as significant. The 24 LD blocks of genetic variants reaching genome-wide significance were obtained from Supplementary Table 8 of Kunkle et al. (2019)^54^. The CSF biomarker-associated genetic loci were obtained from Supplementary Tables 2–4 of Deming et al. (2017)^38^.

### Sensitivity analysis

Immune cell type proportions were estimated using the IDOL algorithm ^55^, as implemented in the estimateCellCounts2 function in the R package FlowSorted.Blood.EPIC. We then fitted the same linear models described in “Identification of CSF biomarker-associated CpGs” above, except by replacing cell type proportions estimated by EpiDISH method with those estimated by IDOL algorithm.

### Validation analysis using an independent dataset

The London dataset^7,56^, which consists of DNAm measured on premortem whole blood samples from 69 subjects, along with their postmortem neurofibrillary tangle burden as measured by AD Braak stage^57^, as well as DNAm measured on the brain prefrontal cortex at autopsy, was downloaded from the GEO database (accession number: GSE29685). The blood and brain DNAm samples from the London dataset were pre-processed in the same way as described above. Given the relatively modest number of samples at some of the Braak stages, we modeled the Braak stage as a binary variable, with absent/low (Braak scores of 0,1,2) vs. intermediate/high (Braak scores 3–6) neurofibrillary tangle tau pathology, as previously described ^28^. Specifically, to test the association between premortem blood DNAm and postmortem AD Braak stage, we fitted the model methylation M value ~ Braak stage (absence/low vs. intermediate/high) + sex + age at blood draw + batch. In the London dataset, none of the estimated blood cell-type proportions were significantly associated with the Braak stage (Supplementary Fig. 1), so they were unlikely to be confounding factors; therefore, we did not include them in the above linear model. To assess concordance between brain and blood DNAm at each CpG within the DMR located on the *HOXA5* gene, we computed Spearman correlations.

## Results

### Sample characteristics

To identify DNA methylation associated with CSF biomarkers, we studied matched whole blood DNA methylation, CSF Aβ_42_, phosphorylated tau_181_ (pTau_181_), and total Tau (tTau) biomarkers data measured on the same subjects and at the same clinical visits in the ADNI study^31,37^. Our study included samples from a total of 202 subjects (123 cognitively normal, 79 AD cases). Table 1 shows the demographic information of these subjects. There were no significant differences in age, sex, smoking history, and educational attainment between the cognitively normal (CN) and AD subjects. Overall, the majority of the subjects are in their seventies (with an average age of 76.6), are highly educated (with an average of 16 years of education), and fewer than half of the subjects smoked. Compared to CN subjects, the AD subjects have a higher proportion of APOE 4 carriers (71% in AD vs. 25% in CN). Moreover, CSF Aβ_42_ levels were significantly lower in AD subjects, while CSF pTau_181_ and tTau levels were significantly higher in AD subjects. Finally, Mini-Mental State Examination (MMSE) scores were significantly lower in AD subjects (an average of 22 points in AD vs. an average of 29 points in CN), indicating more cognitive dysfunction.

#### DNA methylation in the blood is significantly associated with CSF biomarkers at individual CpGs and genomic regions

To identify DNAm differences associated with CSF biomarkers at different stages of the disease, we analyzed CN and AD samples separately. Supplementary Table 3 presents a summary of the significant CpGs and DMRs. In CN samples, after adjusting covariate variables (age, sex, batch effects, years of education, number of *APOE4* alleles, smoking history, immune cell-type proportions), and correcting for genomic inflation in each dataset, we identified 1 CpG cg06171420, located in the vicinity of *PCBP3* gene, significantly associated with CSF levels of total tau (tTau) at 5% false discovery rate (FDR) (Supplementary Table 4). At *P*-value< 1 × 10^− 5^, we identified an additional 34, 15, and 11 CpGs significantly associated with CSF Aβ_42_, pTau_181_, and tTau levels, respectively (Table 2–4, Supplementary Table 4–6). Similarly, the analysis of AD samples revealed 125, 21, and 14 CpGs significantly associated with Aβ_42_, pTau_181_, and tTau at *P*-value < 1 × 10^− 5^, respectively, among which 112, 4, and 3 CpGs also achieved 5% FDR. The greater number of DNAm with significant associations to Aβ_42_ than tau (Supplementary Fig. 2) might be due to CSF Aβ_42_ reduction occurring earlier in the disease process, and thus is associated with more pervasive epigenetic effects.

Among these 198 significant CSF biomarker-associated CpGs in either CN or AD samples, the majority (61% or 120 CpGs) were negatively associated with increased levels of AD biomarkers; about two-thirds were located in distal regions of genes (65% or 129 CpGs); about half of the significant CpGs (51% or 100 CpGs) were located in CpG islands or shores, and only about a third of them were located in gene promoter regions (Supplementary Tables 4–6).

At 5% Sidak adjusted *P*-value, comb-p software identified 81, 18, and 24 differentially methylated regions (DMRs) in CN samples, and 57, 15, and 13 DMRs in AD samples, which were significantly associated with Aβ_42_, pTau_181_, and tTau, respectively (Supplementary Tables 7–9). The number of CpGs in these DMRs ranged from 3 to 23. Among these 184 DMRs that were significant in either CN or AD samples analysis (Supplementary Table 3), about half (58%, 107 DMRs) were negatively associated with increased levels of AD biomarkers; about half of the DMRs (59%, 109 DMRs) were located in promoter regions; and the majority (80% or 147 DMRs) were located in CpG island or shores. Only a very small number of CpGs (16 CpGs), representing 8% of the total significant CpGs, overlapped with a small number of DMRs (14 DMRs) (Supplementary Fig. 3). Interestingly, among the significant CpGs and DMRs, 18% CpGs (36 CpGs) and 32% DMRs (59 DMRs) also overlapped enhancer regions (Supplementary Tables 4–9), which are regulatory DNA sequences that transcription factors bind to activate gene expressions^47,58^.

### Blood DNAm associated with CSF biomarkers differed between diagnosis groups

Overall, we found the DNAm associated with CSF biomarkers were relatively distinct across diagnosis groups. Specifically, there was no overlap between the significant CpGs in AD samples and CN samples (Supplementary Fig. 2). Among the 184 significant DMRs that were significant in either CN or AD sample analysis (Supplementary Table 3), only 3 DMRs (chr15:69744390–69744763, chr6:30130819–30131284, and chr6:30130819–30131362), all of which are CSF Aβ_42_ associated-DMRs, were significant in both CN and AD samples. Consistent with this result, there was only a modest and non-significant correlation between estimated effect sizes of CpG-to-CSF biomarker associations in CN samples vs. those in AD samples among significant CpGs (Spearman ρ = 0.10, 0.06, 0.18 for Aβ_42_, pTau_181_ and tTau-associated CpGs, respectively) (Supplementary Fig. 4). Moreover, our interaction model (Model 2 in Methods), which analyzed the combined CN and AD samples, showed that for the majority of the significant CpGs in CN or AD sample analysis (70% or 139 out of a total of 198 CpGs) (Supplementary Tables 4–9), the DNAm × diagnosis interaction effect was significant, indicating significant different DNAm-to-CSF biomarker associations in the two groups.

#### Pathway analysis revealed DNA methylation associated with CSF biomarkers is enriched in a number of biological pathways in cognitively normal and AD subjects

To better understand biological pathways enriched with significant CSF biomarkers-associated DNA methylation, we next performed pathway analysis using the methylGSA software^48^. At 25% FDR (Methods), a total of 89 and 13 pathways were significant in CN and AD samples, respectively (Supplementary Table 10). Among them, 3 pathways (*calcium signaling pathway, regulation of actin cytoskeleton, neuroactive ligand-receptor interaction*) also reached 5% FDR in CN samples, and 2 pathways (*cardiac conduction* and *muscle contraction*) also reached 5% FDR in AD samples.

We next examined the overlap between significant pathways identified in CN samples and AD samples. Among the 95 pathways that reached 25% FDR in either CN or AD samples, only 7 pathways (7.4%) were significant in both groups (Supplementary Table 10). These seven pathways are *regulation of actin cytoskeleton, neuroactive ligand-receptor interaction, ubiquitin mediated, proteolysis, Wnt signaling pathway, MAPK signaling pathway, cardiac conduction,* and *muscle contraction*. We also found pathway enrichment of the significant CSF biomarker-associated CpGs to be independent in CN samples and AD samples (Supplementary Fig. 5). These pathway analysis results are consistent with those described above for individual CpGs, in which we observed little correlation between estimated effect sizes of CpG-to-CSF biomarkers associations in CN and in AD.

#### Correlation of DNA methylation at significant CSF biomarker-associated CpGs and DMRs with expressions of nearby genes

To prioritize significant DNAm with downstream functional effects, we next correlated DNA methylation levels of the significant DMRs or CpGs with the expression levels of genes found in their vicinity, using matched DNAm and gene expression samples generated from 263 independent subjects (84 AD cases and 179 CN) in the ADNI cohort. In CN subjects, after removing effects of covariate variables in both DNA methylation and gene expression levels separately (Methods), at 5% FDR, we found DNAm at 2 CpGs, and 6 DMRs were significantly associated with target gene expression levels (Supplementary Table 11). Interestingly, aside from 1 CpG (cg14074117) located in the intergenic regions, all CpGs and DMRs were negatively associated with target gene expressions. Among them, 3 DMRs were located in gene promoter regions and negatively associated with expression levels of the target genes at *GSTM5, CAT,* and *CRISP2. GSTM5*belongs to the Glutathione S-Transferase family of genes, which encodes enzymes associated with oxidative stress in neurodegenerative diseases^59,60^. Recently, *GSTM5* was observed to be significantly downregulated in the primary visual cortex brain tissues, an area mildly affected by tau pathology and corresponds to the “early” AD transcriptome ^61^. This previous finding is consistent with our result that DNAm increases with pTau_181_ and tTau levels and are negatively associated with the target gene. Similarly, the *CAT* gene encodes catalase, another key antioxidative enzyme that mitigates oxidative stress^62^. Defects in catalase have been implicated in a number of neurological disorders, including AD^63^.

On the other hand, in AD samples, we found DNAm at 5 CpGs and 5 DMRs were significantly associated with target gene expression levels. Half of these DNAm (4 CpGs and 1 DMR) had a negative correlation with target gene expression. Two DMRs, located in the promoter region of the *TNNT1* gene, were positively associated with the expression level of the *TNNT1* gene, which was shown to be a marker of central nervous system molecular stress associated with neuropsychiatric diseases^64^. Our results are consistent with previous observations that DNAm at some promoter regions is correlated with increased target gene expression^65–68^. While traditionally promoter methylation is thought to be associated with transcriptional silencing by blocking the binding of transcription factors (TFs), which are proteins that bind DNA to facilitate the transcription of DNA into RNA, recent studies suggest more complex patterns of protein – DNA interaction associated with the DNA methylome^69,70^. In particular, several studies observed that the binding and activity of some TFs are enhanced by CpG methylation to activate gene expression ^71–73^. In addition, the positive promoter DNAm to target gene association could also be due to a co-regulatory phenomenon in which both DNAm and target gene are altered by proteins associated with TFs^53,69,74,75^.

### Correlation and overlap with genetic susceptibility loci

To identify methylation quantitative trait loci (mQTLs) for the significant DMRs and CpGs, we next performed look-up analyses using the GoDMC database^53^ for mQTLs. In CN samples, among the 764 individual CpGs or CpGs located within DMRs that are significantly associated with the CSF biomarkers, 301 CpGs had mQTLs in *cis*, and 41 CpGs had mQTLs in *trans*. Similarly, among the 610 significant CpGs or CpGs located in the DMRs in AD samples, 281 and 55 CpGs had mQTLs in *cis* and in *trans*, respectively. Among them, 30127 CpG – mQTL pairs, associated with 16 unique CpGs, were significant in both CN and AD sample analyses (Supplementary Table 12). These results suggested that approximately half of the CSF biomarker-associated CpGs are impacted by genetic variation, consistent with a recent large mQTL meta-analysis of blood samples, which estimated that genetic variants influence about 45% of CpGs on the Illumina array^53^.

Next, to evaluate if the significant mQTLs in CN and AD overlapped with genetic risk loci implicated in AD, we compared the mQTLs with the 24 LD blocks of genetic variants reaching genome-wide significance in a recent meta-analysis of AD GWAS^54^. In CN samples, we found 1518 mQTLs, associated with DNA methylation at 10 significant CpGs (all of which are located in DMRs), overlapped with the LD regions chr 6:32395036–32636434, and 19:1050130–1075979, which included genetic variants mapped to *HLA-DRA, HLA-DRB5, HLA-DRB1, HLA-DQA1, HLA-DQB1* on chromosome 6, and *ABCA7, ARHGAP45, HMHA1* on chromosome 19 (Supplementary Table 13). Similarly, in AD samples, we found 41 mQTLs, associated with DNA methylation at 9 significant CpGs (all of which are located in DMRs), overlapped with the LD regions chr 6:32395036–32636434 and chr 15:58873555–59120077, which included genetic variants mapped to *HLA-DRA, HLA-DRB5, HLA-DRB1, HLA-DQA1, HLA-DQB1* on chromosome 6, and *ADAM10, HSP90AB4P, LOC101928725, FAM63B* on chromosome 15 (Supplementary Table 14). Our comparison of the mQTLs with CSF biomarker-associated genetic loci^38^ did not identify any overlapping variants. These results suggested that the majority of the CSF biomarker-associated CpGs, by and large, are not influenced by genetic variants at the GWAS loci for AD or AD biomarkers. Therefore, even though a substantial proportion of the CpGs are influenced by genetic variants, we found no evidence that genetic variations might be confounding variables in our DNAm to CSF biomarker associations because these genetic variations are not significantly associated with AD or AD biomarkers.

Finally, we also evaluated if our significant methylation loci overlapped with the genetic risk loci associated with AD diagnosis^54^ or CSF AD biomarkers^38^. However, we found no overlap between the significant DNAm discovered in this study compared with AD diagnosis or CSF AD biomarker-associated genetic risk loci. This result is consistent with a previous study which also found no evidence of overlap between significant EWAS loci and GWAS loci in a meta-analysis of 11 blood-based EWAS of neurodegenerative disorders^36^. The lack of commonality between genetic and epigenetic loci in AD supports previous findings that DNA methylation and genetic variants play relatively independent roles in AD^4,76^.

### Sensitivity analysis

We performed an additional analysis to evaluate the robustness of DNAm to CSF biomarker associations with regard to different methods for estimating cell type proportions. To this end, we estimated immune cell type proportions using an alternative method, the IDOL algorithm described in Salas et al. (2018)^55^. Our results show the cell type proportions estimated by the IDOL method and the EpiDISH method^35^ we used in our primary analyses are highly concordant (Supplementary Fig. 6). Next, we repeated our DNAm to CSF biomarkers association analyses by adjusting cell type proportions estimated by IDOL. Our results showed the blood DNAm to CSF biomarker associations obtained by adjusting IDOL cell type proportions are largely congruent with our primary analysis results. In particular, the Aβ_42_- associated CpGs and pTau_8_-associated CpGs remained highly significant, with *P*-values ranging from 1.10 × 10^−10^ to 1.81 × 10^− 4^ (Supplementary Table 15), and 1.39 × 10^− 8^ to 2.92 × 10^− 3^ (Supplementary Table 16), respectively, indicating our results are robust to different algorithms for estimating cell type proportions.

### Validation analysis using an independent dataset

To validate our findings, we also studied DNAm associated with brain pathology in an independent dataset. To this end, we analyzed DNAm measured on premortem blood samples from 69 subjects, along with their postmortem neurofibrillary tangle burden in the brain prefrontal cortex determined at autopsy, as measured by AD Braak stage^57^ in the London dataset ^7 ,56^. At a nominal *P*-value less than 0.05, a number of CSF biomarker-associated CpGs and DMRs that we identified in the ADNI dataset are also significantly associated with the Braak stage in the London dataset (Supplementary Tables 17–18). These DNAm are located at the *ERO1LB, MBTPS1, HOXA5, TRIM15, TYW3, MME, HMSD, CHAD, SEMA3C* genes, and the intergenic regions. Note that because CSF Aβ_42_ decreases and brain tau-pathology increases in AD subjects, we selected CpGs or DMRs with opposite directions in blood DNAm-to-CSF Aβ_42_ and blood DNAm-to-Braak stage associations.

After correcting for multiple comparisons, at Sidak adjusted *P*-value less than 0.05, we observed blood DNAm at two DMRs, located on the *HOXA5* and *CHAD* genes, were significantly associated with AD Braak stage in the London dataset, and overlapped with CSF pTau_181_ or Aβ_42_ associated DMRs in the ADNI dataset. Of particular interest is the strong replication association signal located in the promoter region of the *HOXA5* gene. In ADNI (discovery) dataset, blood DNAm at DMR chr7: 27183946–27184668 is significantly associated with CSF pTau_181_ (*P*-value = 1.06 × 10^− 6^, Sidak-adjusted *P*-value = 1.07×10^− 3^); in London (replication) dataset, blood DNAm at this locus (at DMR chr7: 27183133–27184451) is also significantly associated with Braak stage in the brain (*P*-value = 7.27 × 10^−20^, Sidak-adjusted *P*-value = 2.49 × 10^−17^) (Supplementary Table 18). Previously, Smith et al. (2018) also observed significant hypermethylation across the *HOXA* gene cluster in the brain significantly associated with AD Braak stage in the Mt. Sinai, London, and ROSMAP brain datasets^8^. Intriguingly, we also observed significant correlations between brain and blood DNAm at 7 CpGs located within the DMR (Supplementary Fig. 7), as well as a significant association between the DMR with target gene expression (Supplementary Fig. 8). Together, these results suggested the DMR at *HOXA5* is a promising biomarker robustly associated with tau-pathology in both brain and the blood.

## Discussion

In this study, we analyzed samples from the CN and AD subjects separately, as we reasoned that the CSF biomarker-associated DNAm discovered in CN samples would most likely be associated with AD risk; in contrast, after the onset of disease, the CSF biomarker-associated DNAm in AD samples would most likely be associated with both AD risk as well as changes caused by AD pathologies that accumulate in the brain. Supporting this premise, we found that the significant DNAm identified in AD and CN samples were largely distinct (Supplementary Fig. 2). There was also little correlation between DNAm-to-AD biomarker associations in the two groups of subjects, both at the levels of CpGs (Supplementary Fig. 4) and pathways (Supplementary Fig. 5). These results suggest that the epigenetics associated with different pathological processes in cognitively normal subjects (some of which might later proceed to develop AD) and AD patients vary, supporting the recommendation of considering the patients’ disease stage in developing treatment strategies ^77,78^.

Our comprehensive analyses identified a number of DNAm differences significantly associated with CSF biomarkers Aβ_42_, pTau_181_, and tTau, many of which were associated with genes previously implicated in AD pathogenesis. Specifically, in the analysis of CN subjects, we identified 1 CpG (cg06171420) mapped to around 5 kb upstream of the *PCBP3* gene, significantly associated with tTau at 5% FDR (Supplementary Table 4, Supplementary Fig. 9). The *PCBP3* gene encodes the RNA-binding protein hnRNPE3 (poly(rC) binding protein 3), which regulates alternative splicing of the tau gene^79,80^. In Down Syndrome, AD, and other neurodegenerative diseases, an abnormal ratio of tau protein isoforms often results in aggregated tau, a major component of neurofibrillary tangles. In the region-based analysis, the most significant CSF Aβ_42_-associated DMR is located in the promoter of the *THRB* gene (Supplementary Fig. 10), which encodes a receptor for the thyroid hormone, previously observed to be dysregulated in AD subjects^81–83^.

In AD subjects, we identified significantly more DNA methylation associated with the CSF biomarkers; a total of 112, 4, and 3 CpGs reached 5% FDR in their association with Aβ_42_, pTau_181_ and tTau, respectively. Among the top 10 most significant CpGs associated with Aβ_42_ (Table 2), cg24037493 maps to the promoter of the *SFXN1* gene and is significantly associated with CSF Aβ_42_ in AD subjects (Supplementary Fig. 11). *SFXN1* encodes the mitochondrial serine transporter, which helps to maintain mitochondrial iron homeostasis ^84^. It has been observed that iron levels accumulate in the brains of AD subjects and correlate significantly with cognitive decline^85–87^. Similarly, among the top 10 most significant pTau_181_ and tTau-associated CpGs (Table 3), cg03037740 maps to the promoter of the *RING1* gene, and is significantly associated with CSF pTau_181_ (Supplementary Fig. 12). *RING1* encodes a protein that interacts with the polycomb protein BMI1, which plays a critical role in AD pathogenesis. Remarkably, it has been demonstrated that reduced expression of BMI1 protein alone is sufficient to induce both amyloid and tau pathologies in both cellular and animal models^88,89^. The most significant promoter DMR associated with Aβ_42_ is located at the *TMEM204* gene (Supplementary Fig. 13), which encodes a transmembrane protein that functions as a cell surface marker for infiltrating microglia in the CNS during neuroinflammation^90^. Similarly, the most significant promoter DMR associated with pTau_181_ is located at the *FBP1* gene (Supplementary Fig. 14), which encodes an enzyme that regulates glucose and energy metabolism. It has been observed the expression levels of *FBP1* are reduced in the brains of patients at risk for AD^91,92^, consistent with our observed hypermethylation at the promoter of the *FBP1* gene in samples with increased levels of pTau_181_. Taken together, these results demonstrated that our analysis nominated biologically meaningful DNA methylation loci in the blood associated with AD and, importantly, that changes in the different pathological processes in the CSF, both before and after the clinical diagnosis of AD, are reflected in the epigenome.

In AD samples, the most significant pathways that reached 5% FDR are *cardiac conduction* (*P*-value = 2.76 × 10^− 4^, FDR = 2.54 × 10^− 2^) and *muscle conduction* (*P*-value = 1.42 × 10^− 4^, FDR = 2.54 × 10^− 2^), which also achieved 25% FDR in CN samples (*P*-value = 3.58 × 10^− 4^, FDR = 6.58 × 10^− 2^; *P*-value = 5.63 × 10^− 4^, FDR = 7.85 × 10^− 2^). In recent years, the interaction between the heart and brain has increasingly been recognized^93^. Cardiovascular disease, even subclinical cardiac damage, has been shown to be a significant risk factor for dementia^94–97^.

In CN samples, interestingly, among the most significant pathways enriched with significant CpGs is the KEGG pathway “Alzheimer’s disease”, which was curated based on recent AD literature and included genes that confer AD risks, such as *APOE, PSENEN MAPT, CALM3, MME,* and others. Also, in CN samples, the most significant pathway is the *calcium signaling pathway* (*P*-value = 2.39 × 10^− 4^, FDR = 9.09 × 10^− 3^), consistent with the calcium hypothesis of AD, which posits that dysregulated neuronal calcium homeostasis induces impaired synaptic plasticity, defective neurotransmission, promotes accumulation of Aβ and tau proteins, and subsequently lead to neuronal apoptosis in the brain^98,99^. Moreover, increased levels of free intracellular calcium have also been observed in normal aging, the strongest risk factor for AD^100,101^. The second most significant pathway is the *regulation of actin cytoskeleton* (*P*-value = 1.61 × 10^− 3^, FDR = 2.51 × 10^− 2^), consistent with the observation that synapse degeneration is a key early feature of AD pathogenesis^102,103^, and stability of the actin cytoskeleton is crucial for maintaining functional integrity of the dendritic spines at sites for neurotransmission in the brain^104^. These results suggest that some of the brain impairment during the early stages of the disease (i.e., preclinical) is also reflected in the blood epigenome.

Although the majority of the CSF biomarker-associated DNAm differed in CN and AD samples, our analyses also identified a small number of DMRs that were significantly associated with CSF biomarkers in both groups (Supplementary Fig. 2), which could serve as candidate biomarkers in future studies of AD progression. Specifically, three DMRs, all of which were associated with Aβ_42_, reached Sidak adjusted *P*-value < 0.05 in both CN and AD sample analyses. The first DMR chr15:69744390–69744763 is located at the promoter of the *RPLP1* gene, which encodes a subunit protein of the ribosome. A defective ribosomal function is associated with decreased capacity for protein synthesis, reduced number of synapses, and has been observed as an early feature of AD preceding neuronal loss^105,106^. Another noteworthy result is two overlapping DMRs significantly associated with CSF Aβ_42_, at chr6:30130819–30131284 in AD samples and chr6:30130819–30131362 in CN samples, both are located in the promoter of the *TRIM15* gene, which encodes a member of the TRIM protein family involved in the ubiquitin system responsible for degrading misfolded protein aggregates and plays important roles in neurodegenerative diseases^107,108^.

To validate our findings, we studied premortem blood DNAm associated with postmortem Braak stage measured on prefrontal cortex samples in an independent dataset, previously described as the London dataset^7^. Encouragingly, we found a number of CSF-biomarker-associated blood DNAm also correlated significantly with the Braak stage, which corresponds to neurofibrillary tangle tau pathology burden in the brain (Supplementary Tables 17–18). In the London dataset, we observed a strong blood DNAm to Braak stage association signal located at a DMR in the promoter region of the *HOXA5* gene. Interestingly, this locus also showed a significant association to CSF pTau_181_ in the ADNI dataset (Supplementary Table 18, Supplementary Fig. 15). Moreover, we also observed a significant correlation between brain DNAm and blood DNAm at a subset of 7 CpGs within the DMR (Supplementary Fig. 7), as well as a significant association between the DMR and downstream target gene expression (Supplementary Fig. 8). Consistent with previous studies, which discovered the extensive hypermethylation in the brain at the *HOXA* gene clusters significantly associated with tau neuropathology^7^, our study provided strong evidence that these hypermethylated CpGs can also be observed in the blood epigenome, and are significantly associated with pTau_181_ levels in the CSF (Supplementary Table 18). Taken together, these results nominate hypermethylation at the *HOXA5* locus in the blood as a plausible biomarker for tau pathology.

On the other hand, given brain and blood cells originate from different developmental cell lineages, previous studies also suggested that DNA methylation profiles are, by and large, distinct between brain and blood^7,17,109^. Consistent with these previous results, our comparison of the blood DNAm from this study with brain DNAm associated with AD pathology in two large recent meta-analyses of postmortem brain tissues^9,110^ shows only a few overlapping DNAm (3 CpGs and 8 DMRs), mapped to *PRSSL1, LINGO3, SPRED2, HOXA2, NR2F1, CPT1B, HOXA5, ZFPM1* genes, and intergenic regions, were significant with both blood DNAm-to-CSF Aβ_42_/pTau_181_ association and brain DNAm-to-brain Aβ/tau association (Supplementary Tables 4–9). Also, there is not any overlap between blood DNAm associated with the CSF AD biomarkers and blood DNAm associated with clinical AD from our previous meta-analyses of two large clinical AD datasets^17,111^. This is not surprising, given the disconnection between brain pathology and clinical diagnosis in AD; it has been observed that a substantial proportion of cognitively normal subjects also have AD pathology in the brain^20,21^.

This study has several limitations. First, we analyzed the methylation levels measured on whole blood, which contains a complex mixture of cell types. To reduce confounding effects due to different cell types, we included estimated cell-type proportions as covariate variables in all our analyses. Future studies that utilize single-cell technology for gene expression and DNAm could improve power and shed more light on the particular cell types affected by the DNAm loci discovered in this study. Second, to study DNAm associated with CSF biomarkers in subjects at different stages of the disease (i.e., preclinical or clinical), we separately analyzed samples from cognitively normal and AD subjects, which reduced the sample sizes of the analysis datasets considerably. Given the modest sample size, we pre-defined a more liberal significance threshold (i.e., *P*-value < 10^− 5^) based on previous analyses of blood DNA methylation data ^17,37,43,112^, to select a small number of loci that were then further prioritized using additional integrative analyses. Future studies with larger sample sizes are needed to identify and replicate DNAm loci at more stringent significance thresholds. Third, we did not consider MCI subjects in this study because there is considerable heterogeneity among MCI subjects, with subjects converting to AD at different trajectories^113^. As ADNI is currently conducting additional phases of the study, future analyses with a larger sample size will make it possible to detect DNA methylation to CSF AD biomarker associations in different subgroups of MCI subjects. Fourth, although women make up about two-thirds of AD patients in the general U.S. population^1^, our study cohort (which had both CSF biomarkers and blood DNAm available in ADNI) had a disproportionately lower proportion of females in the AD group (37% females in AD group vs. 51% females in CN group) (Table 1). Therefore, our study cohort may not represent a random sample from the general population. In all our analyses, we adjusted the variable sex in addition to other covariate variables, so the DNAm-to-CSF biomarkers associations we identified are independent of sex. Large and diverse community-based cohort studies that validate our findings are needed. Fifth, as recent autopsy studies revealed that about a quarter of CN subjects also shows AD neuropathology in the brain^20,21^, the CSF biomarker-associated methylation we observed in CN subjects could potentially be markers of an early feature in AD that precedes clinical diagnosis. Future studies that develop DNAm-based prediction models for diagnosing AD and compare their performance with state-of-the-art plasma biomarkers of AD are needed. Finally, the associations we identified do not necessarily reflect causal relationships. Future studies are needed to establish the causality of the nominated DNA methylation markers.

## Conclusions

In this study, we leveraged AD biomarkers as quantitative outcomes to identify DNAm associated with various AD pathology. Our study found a number of novel associations between blood DNAm and CSF Aβ_42_, phosphorylated tau_181_, and total tau, which are proxy biomarkers of AD pathophysiology, demonstrating that changes in various pathological processes in the CSF are reflected in the blood epigenome. Overall, the CSF biomarker-associated DNA methylome is relatively distinct in CN and AD subjects, highlighting the importance of analyzing omics data measured on cognitively normal subjects (which includes preclinical AD subjects) to identify diagnostic biomarkers, and considering disease stages in the development and testing of AD treatment strategies. Our analysis of blood samples of cognitively normal subjects pointed to a number of potential therapeutic targets relevant to the treatment of AD, such as calcium channel blockers associated with *calcium signaling pathway*^98^, and spine stabilizing therapy associated with *regulation of actin cytoskeleton*^104^. Moreover, we found blood DNAm at several CpGs in the DMR on the *HOXA5* gene are not only associated with CSF pTau_181_, but also tau-pathology in the brain, as well as brain DNAm at the same locus in an independent dataset, nominating DNAm at this locus as a promising candidate AD biomarker. In summary, our study provides a valuable resource for future mechanistic and biomarker studies in AD.

## Figures and Tables

**Figure 1:**
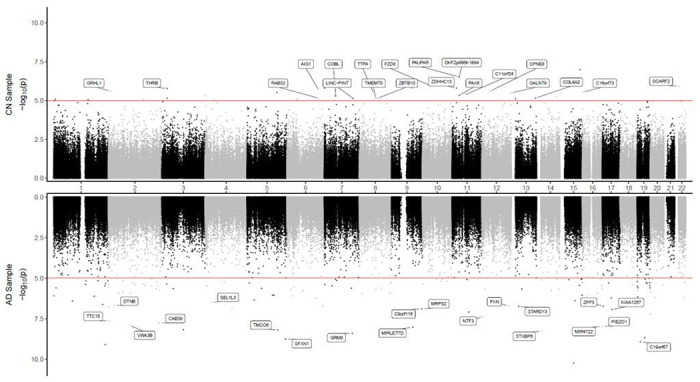
Miami plot for CpGs significantly associated with CSF Aβ_42_ in the ADNI cohort. The X-axis shows chromosome numbers. The Y-axis shows −log_10_ (*P*-value) of methylation-to-CSF Aβ_42_ association in cognitively normal (CN) subjects, or Alzheimer’s disease (AD) subjects. The genes associated with the 20 most significant CpGs per subject group are highlighted. The red line indicates *P*-value < 10^−5^ significance threshold.

**Figure 2:**
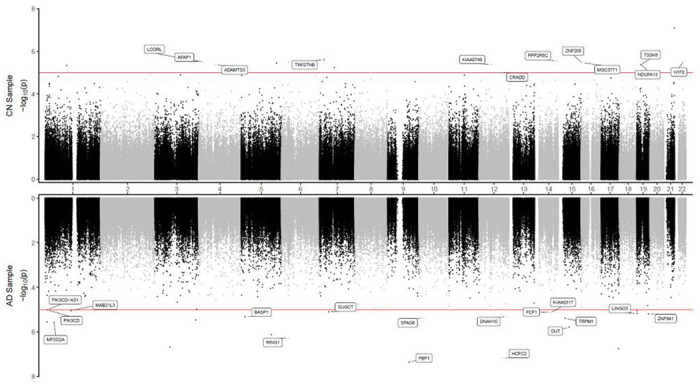
Miami plot for CpGs significantly associated with CSF phosphorylated tau_181_ (pTau_181_) in the ADNI cohort. The X-axis shows chromosome numbers. The Y-axis shows −log_10_ (*P*-value) of methylation-to-CSF pTau_181_ association in cognitively normal (CN) subjects, or Alzheimer’s disease (AD) subjects. The genes associated with the 20 most significant CpGs per subject group are highlighted. The red line indicates the *P*-value < 10^−5^ significance threshold.

**Table 1:** Sample characteristics of the study dataset.

	CN (N = 123)	AD (N = 79)	*P-*value
**N. of females (%)**	63 (51)	29 (37)	0.061
			
**age in year;, mean (sd)**	76.18 (6.62)	77.17 (6.01)	0.217
			
**APOE4 carrier, N (%)**	30 (25)	56 (71)	<,001
			
**smoking, N (%)**	51 (41)	37 (47)	0.544
			
**education in yean, mean(sd)**	16,63 (2.54)	16,11 (2.62)	0,168
			
**CSF Aβ_42_, median (range)**	1332 (318 to 3555)	613 (257 to 3114)	<.001
			
**CSF phosphorylated tau_181_, median (range)**	20 (8 to 63)	33 (10 to 90)	<.001
			
**CSF total tau, median (range)**	231 (112 to 631)	367 (121 lo 370)	<.001
			
**MMSE, mean (sd)**	28.95 (1.34)	21.77 (4,14)	<.001

**Table 2:** Top 10 most significant CpGs associated with CSF Aβ_42_ in cognitively normal (CN) and Alzheimer’s disease (AD) subjects. Annotations include the location of the CpG based on hg19/GRCh37 genomic annotation (chr, position) and nearby genes based on GREAT (GREAT_annotation). Regression analysis results for CpG-to-CSF Aβ_42_ association include effect estimate, standard error (se), and *P*-values after inflation correction using the bacon method (PMID: 28129774). Highlighted in red are gene promoter regions mapped to significant CpGs.

cpg	Annotations			Association with Aβ_42_		
	chr	position	GREAT_annotation	estimate	se	pValue
*sianificant in CN subfects*						
cg01373819	chr15	91209329	BLM (−51229) ;CRTC3 (+136030)	−0.053	0.010	1.03E-07
cg10961330	chr11	31849190	RCN1 (−263260);PAX6 (−9682)	0.064	0.012	3.02E-07
Cg07755l73	chr10	35930525	FZD8 (−164)	−0.373	0.076	1.08E-06
Cg04608146	chr22	20792323	SCARF2 (−178)	−0.119	0.024	1.20E-06
cg21481456	chr9	94351666	NFIL3 (−165523);ROR2 (+360778)	0.050	0.010	1.52E-06
Cg14409029	chr11	19133243	ZDHHC13 (−433)	0.024	0.005	1.58E-06
Cg04116766	chr7	1233255	UNCX (+10713);MICALL2 (+215883)	0,052	0.011	1.58E-06
cg27622506	chr3	24537160	THRB (−895)	−0.108	0.023	1.66E-06
cg17472152	chr6	143436011	AIG1 (+103999);ADAT2 (+285799)	−0.071	0.015	1.94E-06
Cg26510017	chr12	39299546	CPNE8 (−114)	−0.143	0.030	2.46E-06
*significant in AD subjects*						
cg03492603	chr15	62513534	TIN 2 (−340030);C2CD4B (−56053)	−0.188	0.029	5.99E-11
cg07637143	chr1	234333313	IRF2BP2 (−88043);TOMM20 (+458933)	−0.103	0.017	8.39E-10
cg09379609	chr19	14196637	PALM3 (−26667);SAMD1 (+4883)	−0.303	0.050	1.21E-09
cg24037493	chr5	174905263	SFXN1 (−135)	0.076	0.013	1.81E-09
cg16253115	chr19	33781998	SLC7A10 (−65243);CEBPA (+11472)	−0.082	0.014	2.19E-09
cg24700475	chr7	126879927	GRM8 (+13421)	−0.182	0.031	4.07E-09
cg24303533	chr14	25279500	GZMB (−176028);STXBP6 (+240003)	−0.094	0.016	5.47E-09
cg27658391	chr5	140023555	IK (−3800)	−0.079	0.014	6.59E-09
cg25163647	chr3	99113791	DCBLD2 (−493259);COL8A1 (−243528)	−0.070	0.012	6.87E-09
cg03275648	chr9	96940848	ZNF169 (−80745);PTPDC1 (+94103)	−0.080	0.014	9.53E-09

**Table 3:** Top 10 most significant CpGs associated with CSF phosphorylated tau_181_ (pTau_181_) in cognitively normal (CN) and Alzheimer’s disease (AD) subjects. Annotations include the location of the CpG based on hgl9/GRCh37 genomic annotation (chr, position) and nearby genes based on GREAT (GREAT_annotation). Regression analysis results for CpG-to-CSF pTau_181_ association include effect estimate, standard error (se), and *P*-values after inflation correction using the bacon method (PMID: 28129774). Highlighted in red are gene promoter regions mapped to significant CpGs.

cpg	Annotations			Association with pTau_181_		
	chr	position	GREAT_annotation	estimate	se	pValue
*significant in CN subjects*						
cg06171420	chr21	47058388	SLC19A1 (−96004);PCBP3 (−5220)	−0.074	0.014	8.13E-0S
cg20109393	chr7	19748466	TWISTNB (+244)	−0.543	0.115	2.49E-06
cg05020081	chr14	102263662	DYNC1H1 (−167203);PPP2R5C (+35523)	−0.332	0.071	2.82E-06
cg25912009	chr4	7844982	PSAPL1 (−403233);AFAP1 (+96671)	0.144	0.031	3.028-06
cg05568762	chr4	18023126	LCORL (+357)	−0.340	0.073	3.13E-06
cg18001983	chr6	32913125	HLA-DMB (−4279)	−0.054	0.012	3.23E-06
cg01443426	chr22	38201594	GCAT (−2318);H1F0 (+481)	0.074	0.016	3.24E-06
cg27367045	chr16	3165677	ZNF213 (−19352);ZNF205 (+3117)	−0.080	0.017	3.43E-06
cg10015705	chr5	158713704	UBLCP1 (+23616);IL12B (+44191)	−0.063	0.014	3.53E-06
cg07B80109	chr12	55379228	TESPA1 (−773)	−0,029	0.006	4.21E-06
*significant in AD subjects*						
cg14211930	chr9	97402452	FBP1 (+79)	0.086	0.016	4.47E-08
cg14999716	chrl2	104477949	HCFC2 (+19715);NFYB (+54118)	−0.264	0.049	6.81E-08
cg03814390	chr17	77776716	CBX3 (−5802);CBX4 (+36512)	0.346	0.066	1,80E-07
cg01784297	chr3	68716133	FAM19A4 (+265628);FAM19A1 (+662775)	−0.474	0.091	2,10E-07
cg03037740	chr6	33176666	RING1 (+395)	−0.330	0.066	5.42E-07
cg10329462	chr5	135160936	CXCL14 (−245968);SLC25A48 (−9480)	−0.385	0.078	7,57E-07
cg02040583	chr15	48623118	DUT (−502)	−0.120	0.025	1.65E-06
cg15616998	chr1	40435396	CAP1 (−70509);MFSD2A (+14575)	−0.151	0.032	2.64E-06
cg20152585	chr1	8155696	SLC45A1 (−222190);ERRFI1 (−69329)	0.304	0.065	2.86E-06
cg27305835	chr3	184209995	EPHB3 (−69577);CHRD (+112135)	0.153	0.033	3.48E-06

**Table 4:** Top 10 most significant DMRs associated with CSF Aβ_42_ in cognitively normal (CN) and Alzheimer’s disease (AD) subjects. For each DMR, annotations include the location of the DMR based on hg19/GRCh37 genomic annotation (chr, start, end) and nearby genes based on GREAT (GREAT_annotation). Direction indicates a positive or negative association between DNA methylation at a CpG located within the DMR and CSF biomarker. Highlighted in red are gene promoter regions mapped to significant DMRs.

chr	start	end	n_probes	z_p	z_sidak_p	Direction	GREAT_annotation
*(A) significant DMRs in CN subjects*							
3	24536252	24537408	19	1.42E-14	8.98E-12	−−−−−−−−−−−−−−−−−−−	THRB (−564)
6	42927959	42928547	23	2.06E-14	2.55E-11	−−−−−−−−−−−−−−−−−−−−−−−	GNMT (−243)
6	30130819	30131362	10	5.16E-14	6-95E-11	++++++++++	TRIM10 (−2380);TRIM15(+98)
7	142494148	142494596	9	5.91E-13	9.64E-10	+++++++++	EPHB6 (−58420);PRSS1 (+37042)
12	39299326	39299727	7	3.06E-12	5.57E-09	−−−−−−−	CPNE8 (−94)
13	88323992	88324712	7	5.24E-10	5.32E-07	−−−−−−−	SLITRK5 (−518)
3	158449734	158450252	7	1.34E-09	1.89E-06	−−−−−−−	RARRES1 (+492)
2	139537745	139537846	5	2.88E-10	2.08E-06	−−−−−	NXPH2 (+122)
4	30721353	30722153	6	2.41E-09	2.20E-06	−−−−−−	PCDH7 (−1292)
18	25757202	25757711	7	9.86E-09	1.42E-05	−−−−−−−	CDH2 (−47)
*(B) significant DMRs in AD subjects*							
2	3642400	3642968	9	1.89E-17	2.43E-14	+++++++++	COLEC11 (−6812);RPS7 (+19889)
17	41437877	41433403	3	4.76E-14	6.60E-11	+++	ARL4D [−38187];TMEM106A (+74246)
15	91473059	91473570	10	6.15E-13	8.79E-10	−−−−−−−−−−	HDDC3 (+2461);MAN2A2 (+25895)
16	1583391	1584119	10	5.49E-12	5.51E-09	++++++++++	TMEM204 (+181)
15	64673455	64673873	9	4.84E-12	8.46E-09	−−−−−−−−−	KIAA0101 (+45)
17	79004850	79005441	7	7.41E-12	9.16E-09	−−−−−−−	BAIAP2 (−3816)
21	36258423	36259624	11	1.32E-10	8.03E-08	−−−−−−−−−−−	RUNX1 (+162617);CLIC6 (+217336)
6	26224013	26224121	3	1.27E-11	8.61E-08	+++	HIST1H3E (−1316)
20	57607406	57607693	8	4.50E-11	1.15E-07	++++++++	ATP5E (−113)
12	120652604	120652845	4	457E-11	1.39E-07	−−−−	RPLPO (−13823);PXN (+50838)

**Table 5:** Top 10 most significant DMRs associated with CSF phosphorylated tau_181_ (pTau_181_) in cognitively normal (CN) subjects and Alzheimer’s disease (AD) subjects. For each DMR, annotations include the location of the DMR based on hgl9/GRCh37 genomic annotation (chr, start, end), and nearby genes based on GREAT (GREAT_annotation). Direction indicates a positive or negative association between DNA methylation at a CpG located within the DMR and CSF biomarker. Highlighted in red are gene promoter regions mapped to significant DMRs.

chr	start	end	n_probes	z_p	z_sidak_p	Direction	GREAT_annotation
*(A) significant DMRs in CN subjects*							
1	19110562	19110979	5	3.05E-11	5.34E-08	−−−−−	TAS1R2 [+75405);PAX71 (+153271)
18	59997009	59997176	5	6.51E-11	2.85E-07	−−−−−	ZCCHC2 (−193147);TNFRSF11A (+4545)
11	64703192	6470363B	8	3.07E-10	5.03E-7	−−−−−−−−	GPHA2 (−55)
15	91473167	91473570	7	8.70E-08	1.58E-04	+++++++	HDDC3 (+2407);MAN2A2 (+25949)
1	226362309	228362510	3	1-09E-07	3.98E-04	−−−	OBSCN (−31421);IBA57 (+8394)
1	110254709	110254897	5	4.57E-07	1.78E-03	+++++	GSTM5 (−74)
2	198650985	198651224	6	6.94E-07	2.12E-03	++++++	BOLL(+193)
21	44898090	44898207	4	5.11E-07	3.19E-03	++++	SIK1 (−51141);HSF2BP (+181225)
6	49681178-	49681392	8	1.02E-06	3.49E-03	−−−−−−−−	CRISP2 (−11)
13	111522222	111522315	3	5.59E-07	4.38E-03	+++	ANKRD10(+45147);ING1 (+154940)
*(B) significant DMRs in AD subjects*							
4	174203061	174203521	5	5.23E-12	5.31E-09	−−−−−	HMGB2 (+52985);GALNT7 (+113387)
9	97402452	97402658	3	6.03E-10	2.14E-06	+++	FBP1 (−24)
17	7311742	7312082	5	4.20E-08	9.02E-05	+++++	TMEM256-PLSCR3 (−4496);TMEM256 [−4456);NLGN2 (+410)
19	55660514	55660659	6	7.39E-08	1.57E-04	−−−−−−	TNNT1 [+35)
16	8961250	8961472	6	5.25E-08	1.73E-04	++++++	CARHSP1 (+897)
3	10149803	10149980	5	2.26E-07	9.33E-04	+++++	BRK1(−7384);FANCD2 [+81794)
7	27183946	27184663	18	1.06E-06	1.07E-03	++++++++++++++++++	HOXA5 (−1020)
5	102898463	102898730	6	5.34E-07	1.46E-03	−−−−−−	NUDT12 (−103)
1	204183116	204183582	7	4.74E-06	7.41E-03	+++++++	GOLT1A [−129)
10	134226361	134226548	3	2.06E-06	8.02E-03	+++	INPP5A (−124869);PWWP2B (+15783)

## Data Availability

The ADNI can be accessed from http://adni.loni.usc.edu The scripts for the analysis performed in this study can be accessed at https://github.com/TransBioInfoLab/AD-ATN-biomarkers-and-DNAm
